# Eastward Jet Lag is Associated with Impaired Performance and Game Outcome in the National Basketball Association

**DOI:** 10.3389/fphys.2022.892681

**Published:** 2022-06-16

**Authors:** Josh Leota, Daniel Hoffman, Mark É. Czeisler, Luis Mascaro, Sean P.A. Drummond, Clare Anderson, Shantha M.W. Rajaratnam, Elise R. Facer-Childs

**Affiliations:** ^1^ Turner Institute for Brain and Mental Health, School of Psychological Sciences, Monash University, Melbourne, VIC, Australia; ^2^ St Kilda Football Club, Australian Football League, Melbourne, VIC, Australia; ^3^ Institute for Breathing and Sleep, Melbourne, VIC, Australia; ^4^ Department of Psychiatry, Brigham and Women’s Hospital, Boston, MA, United States; ^5^ Harvard Medical School, Boston, MA, United States; ^6^ Departments of Medicine and Neurology, Division of Sleep and Circadian Disorders, Brigham and Women’s Hospital, Boston, MA, United States; ^7^ Division of Sleep Medicine, Harvard Medical School, Boston, MA, United States; ^8^ Danny Frawley Centre for Health and Wellbeing, Moorabbin, VIC, Australia

**Keywords:** circadian disruption, elite athletes, sport, sleep, travel, NBA, time zone, phase advance

## Abstract

**Objectives:** Elite athletes are often required to travel across time zones for national and international competitions, causing frequent jet lag. The aim of this study was to examine whether the direction of travel-related jet lag is associated with performance in the National Basketball Association (NBA), and if so, to explore potential mechanisms.

**Methods:** Ten seasons comprising of 11,481 games of NBA data from the 2011/2012 to the 2020/2021 regular season were analyzed using multi-level mixed models with one fixed factor (three levels; jet lag direction: eastward vs westward vs no jet lag) and three random factors (team, opponent, game time). Predicted circadian resynchronization rate was accounted for, and home and away games were analysed separately. Mediation analyses were performed to examine potential mechanisms.

**Results:** Among home teams, eastward (but not westward) jet lag was associated with reduced winning (Δ (i.e., change) = −6.03%, *p* = 0.051, marginal), points differential (Δ = −1.29 points, *p* = 0.015), rebound differential (Δ = −1.29 rebounds, *p* < 0.0001), and effective field goal percentage differential (Δ = −1.2%, *p* < 0.01). As the magnitude of eastward jet lag increased, home team points differential decreased (2 h Δ = −4.53 points, *p <* 0*.*05; 1 h Δ = −0.72 points, *p* = 0.07). No significant associations were found between jet lag and away team performance.

**Conclusion:** Eastward jet lag was associated with impaired performance for home (but not away) teams. Sleep and circadian disruption associated with advancing phase following eastward travel may have significant adverse consequences on performance in the NBA, particularly when recovery time is limited. Sports organisations could consider chronobiology-informed scheduling and interventions to maximise recovery and performance of their athletes.

## Introduction

Optimal athletic performance is critical to success in elite sport (Tucker and Collins, 2012). High performance programs are carefully planned considering training loads, injury reduction and sleep due to the overwhelming evidence of their influence on performance (Fullagar et al., 2015; Fox et al., 2018; Hoffman et al., 2020). Travel is also a key consideration as, when coupled with a condensed competition schedule allowing insufficient time for recovery and adjustment, frequent air travel can lead to fatigue and circadian disruption due to a mismatching of internal biological rhythms with a new time zone (i.e., jet lag), both of which are detrimental to athletic recovery and performance (Singh et al., 2021). While the general effects of travel have been studied extensively, the specific effects of east-west travel across time zones on team performance remains a matter of debate (Leatherwood and Dragoo, 2013).

### Eastward and Westward Travel

Endogenously driven, circadian rhythms govern a wide range of physiological processes involved in athletic recovery and performance (Facer-Childs and Brandstaetter, 2015; Simmons et al., 2022). These rhythms are synchronized to the 24-h environment via the light-dark cycle and behavioral cycles such as eating or drinking (Stephan, 2002; Roenneberg et al., 2013; Asher and Sassone-Corsi, 2015; Wehrens et al., 2017; Lewis et al., 2018). Travel across time zones can desynchronize internal circadian rhythms from each other and from external environmental time cues, causing disruptions to athlete performance, health, and well-being (Manfredini et al., 1998; Waterhouse et al., 2002; Janse van Rensburg et al., 2020). This transient circadian misalignment and its associated consequences—termed “desynchronosis”, or colloquially “jet lag”—last until internal circadian rhythms entrain to the 24-h environment of the new destination, which typically occurs at a rate of approximately 1 hour per day (Aschoff et al., 1975; Takahashi et al., 1999; Song et al., 2017; Clements et al., 2021). Although athletes exhibit a wide range of jet lag symptomology following rapid time zone travel (e.g., daytime sleepiness, sleep disruption, lapses in concentration and motivation, physical fatigue; Leatherwood and Dragoo, 2013), performance has been found to return to normal levels after circadian resynchronization following travel (Chapman et al., 2012).

Chronobiological models of rapid travel have generated conflicting results and associated hypotheses about which *direction* of travel is more detrimental to athletic performance. These models can be organized into two generalized views; 1) the asymmetrical jet lag hypothesis posits that eastward travel is more disadvantageous, while 2) the optimal timing hypothesis posits that westward travel is more disadvantageous. Reconciling these opposing views is necessary to not only optimize athlete performance and health, but to also ensure competitive equality in leagues that require frequent and rapid travel.

### Asymmetrical Jet Lag Hypothesis: An Eastward Disadvantage

Estimates of the endogenous, free-running human rhythm range from 23.8 to 24.6 h (Loat and Rhodes, 1989; Czeisler et al., 1999), with most slightly longer than the 24-h day. Research on both humans and animal models have shown that it is typically more difficult to adjust to a shorter day (i.e., phase advance) than a longer day (i.e., phase delay) (Yamazaki et al., 2000; Burgess et al., 2003). Eastward travel—where the destination time is later than the origin time—requires the athlete to shorten their day (phase advance). Westward travel, on the other hand, allows the athlete to lengthen their day (phase delay). During phase advance, athletes often struggle to fall asleep at an earlier bedtime, leading to sleep loss (Waterhouse et al., 1997; Song et al., 2017) and, consequently, impaired physiological performance and motivation the next day (Fowler et al., 2017). Empirical studies have shown deleterious effects of eastward travel on athlete and team performance. For example, eastward travel is negatively associated with winning percentage in Major League Baseball (MLB) (Recht et al., 1995; Song et al., 2017), points differential in College Football (Worthen and Wade, 1999), team rankings and player physical and technical performance in International Soccer (Zacharko et al., 2020), and lower body muscle performance in elite skeleton athletes (Chapman et al., 2012).

### Optimal Timing Hypothesis: A Westward Disadvantage

In stark contrast to the asymmetrical jet lag hypothesis, some studies report a westward travel disadvantage, citing a mismatch between game times and the athlete’s optimal physiological timing window. Evidence suggests athletes generally perform at their physical best in the late afternoon to early evening (∼16:00–20:00 h; Reilly and Waterhouse, 2009). This optimal performance window is consistent with the rhythm of resting core body temperature (Arnett, 2002) and mirrors diurnal variability in muscle strength, flexibility, and short-term high-power output (Atkinson and Reilly, 1996; Reilly et al., 2000; Drust et al., 2005). Rapid westward travel before a night game could disadvantage athletes, as they are required to perform further from their internal optimal timing window and closer to when their internal circadian system begins to promote sleep. In contrast, rapid eastward travel before a night game may benefit athletes by shifting the game time closer to their optimal timing window. Some empirical research supports the optimal timing hypothesis. For example, westward travel was negatively associated with winning percentages in the National Basketball Association (NBA) (McHill and Chinoy, 2020), National Hockey League (NHL), and National Football League (NFL) (Roy and Forest, 2018) and eastward travel was positively associated with winning percentages in MLB (Winter et al., 2009). However, individual variability in circadian phenotype has been shown to result in different diurnal physiological and performance profiles, which may skew results that do not account for these differences (Facer-Childs and Brandstaetter, 2015; Facer-Childs et al., 2018).

### The Current Research

With 82 regular-season games per team each year over approximately 180 days, including 41 away games requiring travel to and from opposing teams’ arenas, NBA teams are constantly impacted by rapid travel across time zones, causing frequent jet lag and travel fatigue (Singh et al., 2021). However, as reviewed above, the impact of such jet lag remains unclear. Conflicting findings in the literature have been further complicated by widespread methodological and data analytical differences across studies. To reconcile this literature, it is critical to account for potential confounding variables, including team quality, recovery time between games (i.e., allowing for circadian resynchronization), game time, and factors related to playing in home environments. Here, we account for such variables in an analysis of 10 regular seasons of NBA data (2011/2012 to 2020/2021). Our first aim was to examine whether the direction of travel-related jet lag is associated with game outcome and points differential in NBA regular-season games. Our second aim was to examine potential mechanisms (i.e., team performance variables) of eastward or westward jet lag on game outcome.

## Materials and Methods

Ten seasons of NBA game data from the 2011/2012 to the 2020/2021 regular (non-playoffs) seasons were scraped from the open access official statistics partner of the NBA (www.Basketball-Reference.com; Sports Reference LLC, 2017). Team franchises were treated as being constant during these 10 seasons, therefore where name and/or location changes occurred, teams were recoded to their 2020/2021 season name. Consequently the New Jersey Nets, Charlotte Bobcats and New Orleans Hornets were recoded to the Brooklyn Nets, Charlotte Hornets, and New Orleans Pelicans, respectively. Game locations affected by daylight saving time were accounted for during time zone calculations. Games directly following off- and pre-seasons were excluded as travel details prior to the season were unknown. Further, Orlando bubble 2019/2020 season games were excluded due to the removal of travel between games (Haislop, 2020). Therefore, a total of 11,481 games played by 30 teams met the inclusion criteria.

Data analyses were performed in R version 4.0.2 (R Core Team, Vienna, Austria). Multi-level mixed linear models were used with one fixed factor (three levels; jet lag direction: eastward vs westward vs no jet lag) and three random factors (team, opponent and game time) to control for team and opponent quality, and game time. Note that home teams can experience jet lag when returning home from an away game. Home and away games were analyzed separately to control for confounding factors associated with game location (e.g., home crowds; Leota et al., 2021), for ease of interpretation and visualization of data, and to examine whether travel direction impacts home and away teams differently, particularly given that away teams experienced jet lag more than home teams (Table 1).

**TABLE 1:** Directional Jet Lag Occurrences (Games) for Home and Away Teams.

Directional Jet Lag	Home	Away
No Jet Lag	10,411	9,583
Westward Jet Lag	619	928
Eastward Jet Lag	451	970
Total	11,481	11,481

Eastward or Westward Jet Lag is determined as the number of time zones travelled after accounting for circadian resynchronization of 1 h (or 1 time zone) per day

### Directional Jet Lag

**FIGURE 1:**
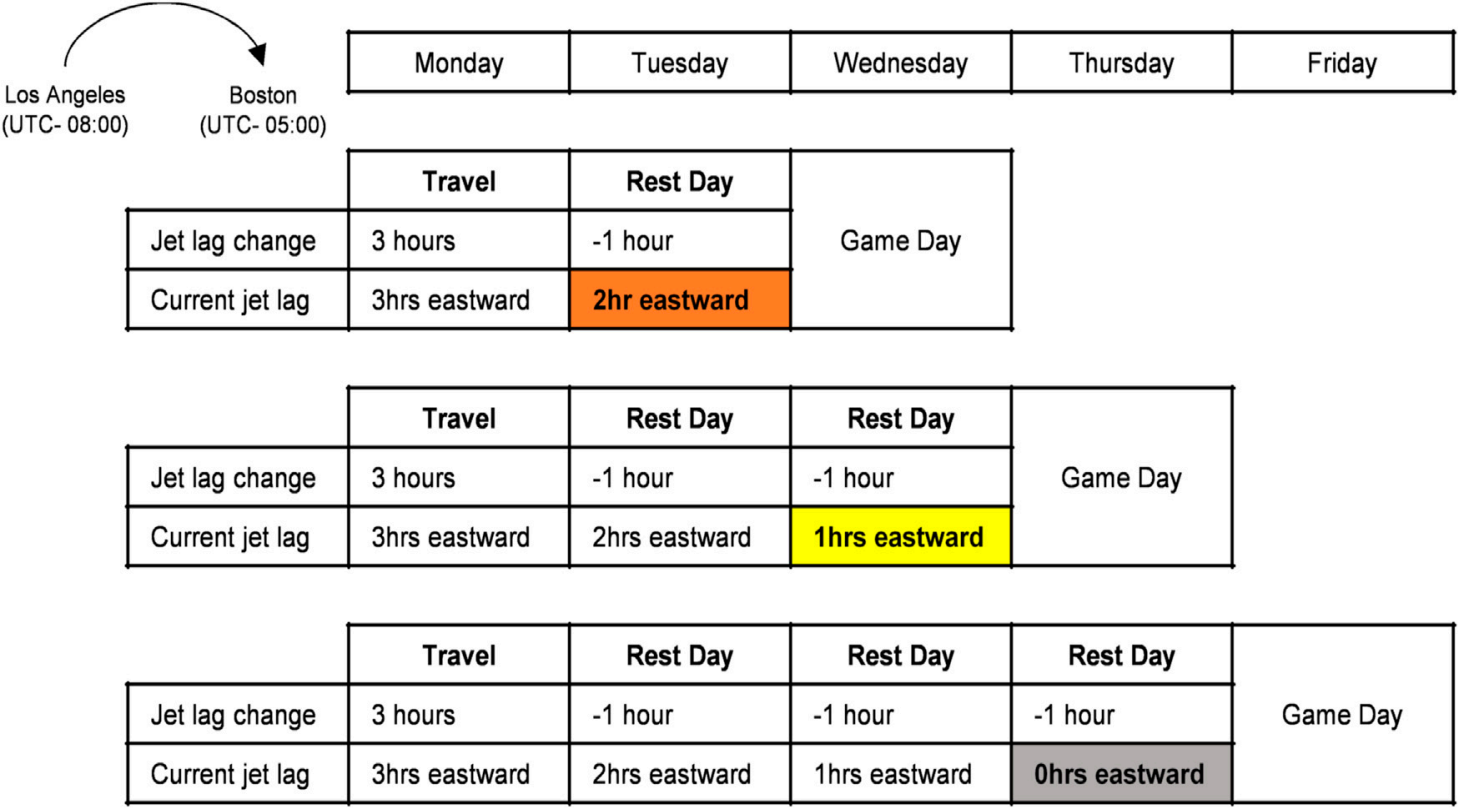
A visual representation of how jet lag was estimated based on game dates and locations. For example, Team A travels from Los Angeles (PST; UTC−08:00) to Boston (EST; UTC−05:00) on Monday, has a rest day on Tuesday, then plays Team B on Wednesday. Team A would be assigned a value of 2 h eastward jet lag for the game against Team B (3 h eastward travel minus 1 h resynchronization) and therefore be classified in the eastward jet lag category.

As athletes have been found to return to baseline performance levels following circadian resynchronization, jet lag was determined as the number of time zones travelled after accounting for a resynchronization rate of 1 hour per day (Winter et al., 2009; Song et al., 2017; see Figure 1 for an example and Supplementary Material S1 for a randomly selected example of one month of an NBA team’s game schedule)). Jet lag effects are evident in athletes after time zone shifts of just 1 h (Loat and Rhodes, 1989). Therefore, teams that experienced 1 h or more jet lag were grouped based on the travel direction (eastward or westward) and all other games were grouped as no jet lag (Table 1). Note that there were no instances of 3 h jet lag (i.e., no games immediately following travel across 3 time zones). Where significant directional associations existed, we also examined 1) whether the magnitude of jet lag was a significant factor (e.g., comparison of no jet lag to 1 h eastward jet lag and 2 h eastward jet lag if a significant eastward directional finding existed) and 2) whether significant directional associations disappeared when teams were given an adequate recovery window to allow for circadian resynchronization.

### Performance Variables

To measure game outcome and performance (Aim 1), we examined winning percentage (1 = win, 0 = loss), points differential (team points minus opponent points) and net rating (points differential per 100 possessions). Next, we examined associations between directional jet lag and two proposed mechanisms of the Asymmetrical Jet Lag Hypothesis: impaired motor performance and effort (Aim 2).^1^ Eastward travel requires a phase advance which is associated with greater circadian disruption and sleep loss. Consistent with past research (Leatherwood and Dragoo, 2013; Fowler et al., 2017), we reasoned that circadian disruption and sleep loss may be associated with impaired motor performance and effort. To measure motor performance, we examined effective field goal percentage (eFG%) differential. eFG% tracks team shooting accuracy adjusted for the fact that 3-point field goals are worth more than 2-point field goals. To measure effort, we examined rebounding differentials (offensive, defensive and total). Rebounding (retrieving the ball after a missed field goal) is considered an effort or hustle play as it entails fighting for position under the hoop and can often lead to physical contact (Maheswaran et al., 2012; White and Sheldon, 2014; Leota et al., 2021). Finally, mediation analyses bootstrapped to 1,000 simulations were performed to determine if potential associations between directional jet lag and points differential^2^ were mediated by eFG% differential or rebounding differentials.

## Results

**FIGURE 2:**
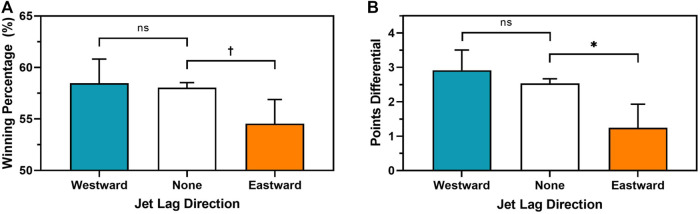
Raw means based on the association between directional jet lag (blue = westward, white = none (reference), orange = eastward), and home team **(A)** winning percentage; and **(B)** points differential. ns = not significant, *p* > 0.10, **†** *p* < 0.07, * *p <* 0.05.

We first examined whether jet lag was associated with game outcome and points differential (Aim 1).^3^ Home teams playing with eastward jet lag (*M* = 54.55%, SD = 0.50) won marginally less than home teams playing with no jet lag (*M* = 58.05%, *SD* = 0.49; *t* = −1.955, *p* = 0.051; Figure 2A). In contrast, home teams playing with westward jet lag (*M* = 58.48%, SD = 0.49) won at a similar rate to home teams playing with no jet lag (*t* = 0.433, *p* = 0.665). Similarly, home teams playing with eastward jet lag (*M* = +1.24, SD = 14.57) had a worse points differential than home teams playing with no jet lag (M = +2.53, SD = 13.70; *t* = -2.441, *p* = 0.015; Figure 2B). Again, home teams playing with westward jet lag (M = +2.91, SD = 14.64) had a similar points differential to home teams playing with no jet lag (*t* = 0.711, *p* = 0.477). Results remain significant after adjusting for number of possessions (i.e., net rating, *p* < 0.05). There were no statistically significant directional jet lag effects on game outcome (*p values* > 0.136) or points differential (*p values* > 0.294) for away games.

**FIGURE 3:**
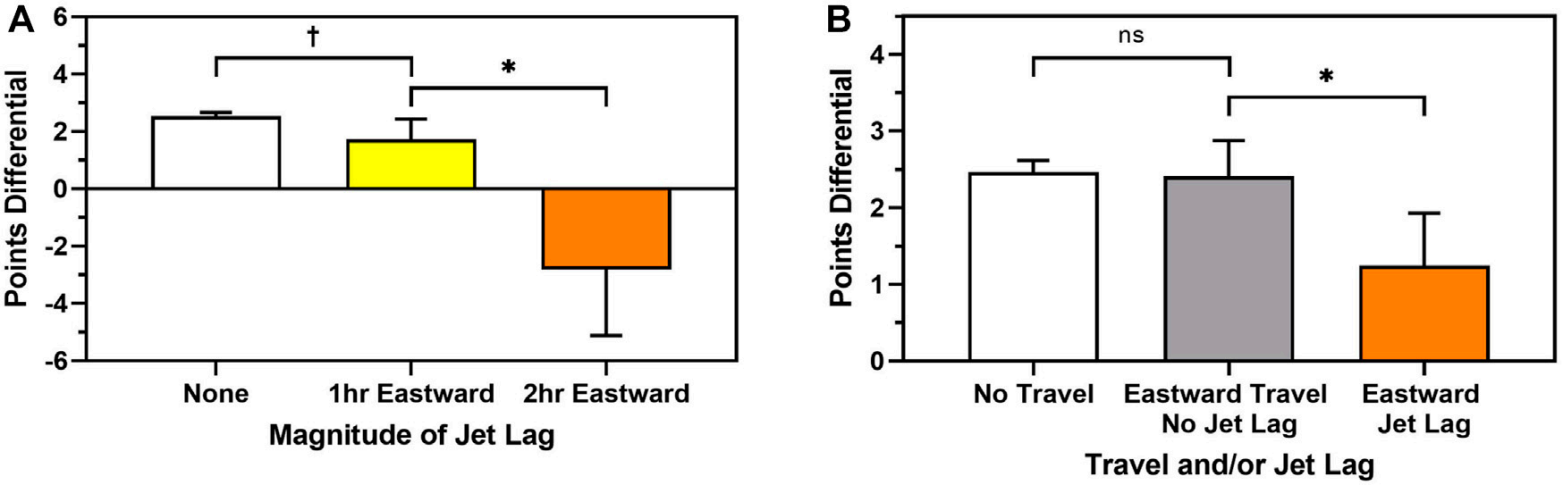
Raw means based on the association between **(A)** the magnitude of eastward jet lag (white = none, yellow = 1h (reference), orange = 2h) and home team points differential, and **(B)** eastward travel and jet lag combinations (white = no travel, grey = eastward travel and no jet lag, orange, eastward jet lag) and home team points differential. ns = not significant, *p* > 0.10, **†** *p* < 0.07, * *p <* 0.05.

Next, we examined whether the *magnitude* of eastward jet lag was associated with points differential for home teams. Home teams playing with 2 h eastward jet lag (*M* = −2.81, SD = 15.98) had a worse points differential than home teams playing with 1 h eastward jet lag (*M* = +1.72, SD = 14.36, *t* = −2.445, *p* = 0.014), who then had a marginally worse points differential than home teams playing with no jet lag (M = +2.53, SD = 13.70; *t* = -1.812, *p* = 0.070; Figure 3A).

Next, we examined whether eastward travel *regardless* of jet lag was associated with points differential for home teams. Home teams playing with eastward jet lag (M = +1.24, SD = 14.57) had a worse points differential than home teams who had travelled eastward but had no jet lag (i.e., had adequate days between games to resynchronize to the new time zone; M = +2.41, SD = 13.81; *t* = −2.463, *p* = 0.014). In contrast, home teams who had travelled eastward but had no jet lag had a similar points differential to home teams who had not travelled at all (M = +2.47, SD = 13.59; *t* = −0.02, *p* = 0.984; Figure 3B). Results are similar with outcome as the dependent variable.

**FIGURE 4:**
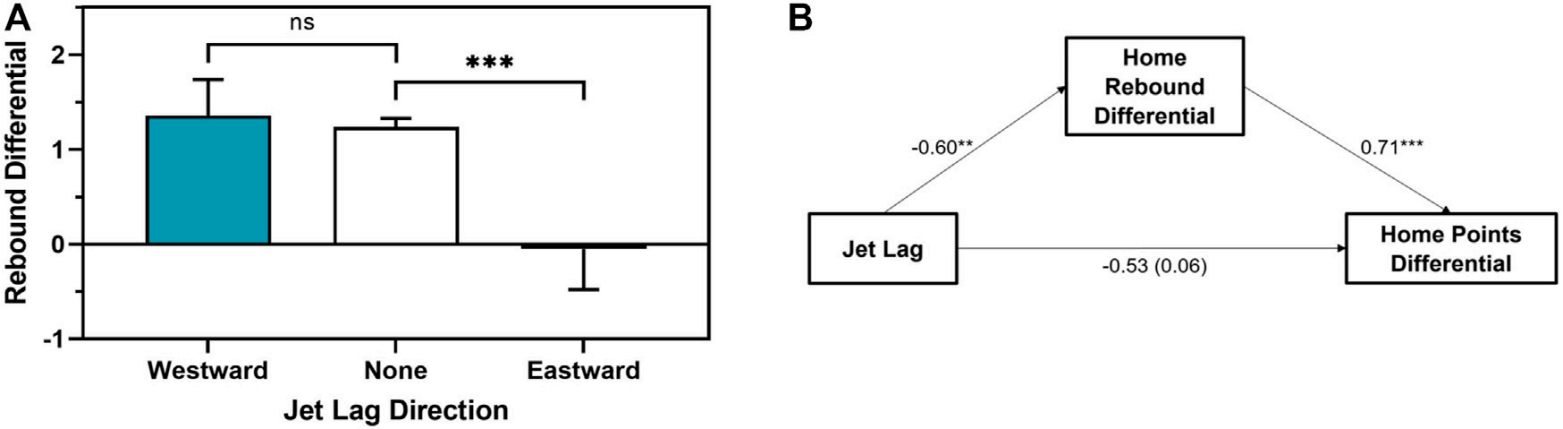
**(A)** represents raw means based on the association between directional jet lag (blue = westward, white = none (reference), orange = eastward) and home team total rebound differential. **(B)** represents the mediational model and regression coefficients for the relationship between directional jet lag and home points differential as mediated by home rebound differential. The regression coefficient between jet lag and home points differential, controlling for home rebound differential, is in parentheses. ns = not significant*, p* > 0.10, ** *p* < 0.01, *** *p* < 0.001.

**FIGURE 5:**
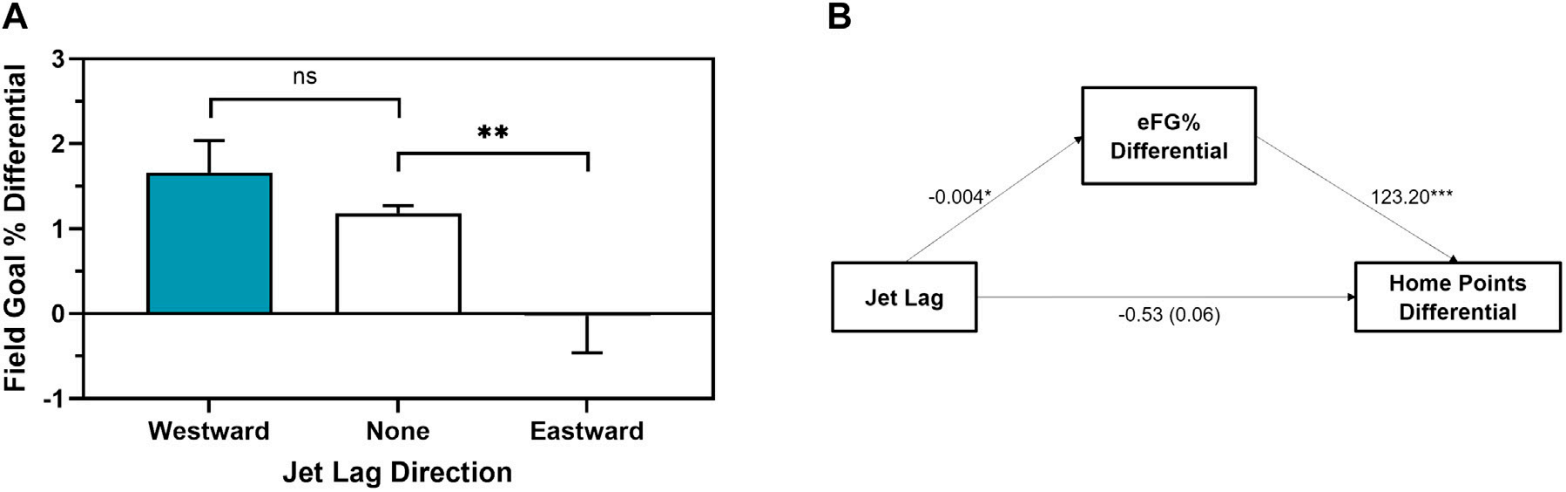
**(A)** represents raw means based on the association between directional jet lag (blue = westward, white = none (reference), orange = eastward) and home team effective field goal percentage (eFG%) differential. **(B)** represents the mediational model and regression coefficients for the relationship between directional jet lag and home points differential as mediated by home eFG% differential. The regression coefficient between jet lag and home points differential, controlling for home eFG% differential, is in parentheses. ns = not significant, *p* > 0.10, *p < 0.05, **p < 0.01, ***p < 0.001.

Finally, we examined the relationship between jet lag direction and potential mechanisms of the *Asymmetrical Jet Lag Hypothesis*, which was the only hypothesis supported by the previous analyses (Aim 2). Home teams playing with eastward jet lag (*M* = −0.05, SD = 9.08) had a worse total rebound differential than home teams playing with no jet lag (M = +1.24, SD = 9.05; *t* = −3.376, *p* < 0.001; Figure 4A). In contrast, home teams playing with westward jet lag (M = +1.36, SD = 9.42) had a similar total rebound differential to home teams playing with no jet lag (*t* = −0.188, *p* = 0.851). Further, total rebound differential mediated the association between home team eastward jet lag and points differential (95% CI −0.71 to −0.16, *p* < 0.001; Figure 3B). Home teams playing with eastward jet lag (*M* = -0.02%, SD = 9.3%) also had a worse eFG% differential than home teams playing with no jet lag (M = +1.2%, SD = 9.0%; *t* = −2.848, *p* = 0.004; Figure 5A). In contrast, home teams playing with westward jet lag (M = +1.7%, SD = 9.4%) had a similar eFG% differential to home teams playing with no jet lag (*t* = 0.946, *p* = 0.344). Further, eFG% differential mediated the association between home team eastward jet lag and points differential (95% CI −0.96 to −0.07, *p* = 0.028; Figure 5B).

## Discussion

Our findings from this analysis of 11,481 regular-season NBA games spanning the 2011/2012 through 2020/2021 seasons support the asymmetrical jet lag hypothesis, at least as it applies to home teams. When playing with eastward jet lag, home teams won fewer games (this association was marginal, *p* = 0.051) and had a worse points differential than when playing with no jet lag. Expressed as a percentage, eastward jet lag was associated with a 6.03% decrease in home team winning percentage, the equivalent of 2.47 fewer home wins over a full NBA regular season (41 home games). This disadvantage increased as the magnitude of eastward jet lag increased, to the point where home teams playing with 2 h eastward jet lag had a negative points differential of almost three points, despite the robust and well-documented advantages typically associated with playing at home (e.g., home crowds, venue familiarity and opponent travel; Ribeiro et al., 2016; Harris and Roebber, 2019; Leota et al., 2021). Further, the eastward disadvantage disappeared when eastward travel was compensated with an adequate recovery window. Differences in winning percentage and points differential were not found for home teams with westward jet lag. The observed eastward jet lag disadvantage for home teams only is consistent with findings from elite baseball (Song et al., 2017).

The eastward jet lag disadvantage experienced by home teams could be explained by worse eFG% differential (i.e., impaired shooting) and worse total rebound margin (i.e., reduced effort). Difficulties in advancing the timing of the circadian clock required following rapid eastward travel may lead to sleep restriction, which has been shown to negatively impact NBA athletes’ motor performance and motivation (Walsh et al., 2021). These findings complement research on insufficient sleep and performance in the NBA. For example, late-night tweeting (which was used as a proxy for sleep deprivation) was associated with worse shooting accuracy and fewer rebounds (Jones et al., 2019). While it is important to acknowledge that coarse-grained data like eastward jet lag and late-night social media behavior do not directly capture individual athlete sleep and circadian variables, these studies are critical for stimulating future research in this area.

NBA athletes may have a later optimal timing window compared to athletes from other sports. Given that most NBA games (>90%) are played between 18:00 and 21:30 h, NBA athletes are incentivised to be at their best later in the day. A delayed optimal timing window may be a consequence of delayed sleep-wake timings due to night games and late-night travel (Singh et al., 2021) or pregame naps (Barnes, 2020). This may account, in part, for why we did not find evidence of a westward travel disadvantage in our data. Consistent with our hypothesis that NBA players have a delayed performance window, Nutting and Price (2017) found no significant relationship between travel and visiting team win probability among night games from 1991 to 2013. We note that we did not measure individual circadian rhythms and can therefore only speculate about performance timings. Future work should investigate diurnal variations in performance among NBA (or other) athletes and take individual differences into account where possible. Indeed, the optimal performance window for any given sport depends not only on the athletes’ diurnal patterns but also the type of activities most critical to overall performance (Reilly et al., 2000). For example, strength-based performance typically peaks in the late afternoon and early evening (Reilly et al., 2000; Seo et al., 2013), but cognitive performance can peak earlier in the day (Colquhoun, 1972; Schmidt et al., 2007). Consequently, the optimal timings for sports that involve multiple psychological and physiological elements—such as NBA basketball—are complex and remain unclear (Drust et al., 2005).

In line with past studies on jet lag in professional sports (Song et al., 2017), eastward jet lag was not associated with away team performance. This could be explained by the controlled environment of travelling NBA teams. Team management could mitigate jet lag effects by maintaining a structured schedule (e.g., transportation, meal timings, athlete treatment sessions, and trainings) while on the road compared to when athletes travel to their individual homes and attend to familial and social obligations (Singh et al., 2021). Another possibility is an away game floor effect due to other factors, such as opposing crowds. For example, in a recent study analysing games from the 2020/2021 NBA season, we found that opposing crowds were associated with a 16% reduction in away team winning percentage (Leota et al., 2021). It is possible that away teams are already sufficiently disadvantaged that the added impact of eastward jet lag is difficult to identify (Song et al., 2017).

The impact of eastward jet lag on circadian desynchrony and insufficient sleep poses a significant challenge to competitive balance and schedule equality in the NBA. An eastward travel disadvantage is of particular concern to NBA schedule equality, as teams are affected disproportionately depending on their geographical location, with teams located on the east coast disproportionately exposed to home games with eastward jet lag. Schedulers could mitigate these effects by compensating eastward travel with increased recovery time to allow athletes to resynchronize to the new time zone (Eastman and Burgess, 2009). Indeed, our data show that when eastward travel was followed by an adequate recovery window, home teams performed similarly to when they did not travel at all. Allowing time for the circadian system to realign naturally to the destination’s light-dark cycle could, therefore, mitigate the observed eastward travel disadvantage.

If schedule changes to allow for natural circadian resynchronization are deemed unfeasible by the NBA, teams could implement evidence-based interventions to manage eastward jet lag, including specifically timed light exposure and avoidance (Roach and Sargent, 2019), exercise (Youngstedt et al., 2019), or exogenous melatonin supplementation (Burgess et al., 2010; Burke et al., 2013). Importantly, a recent review (Janse van Rensburg et al., 2020) and consensus statement (Janse van Rensburg et al., 2021) concluded that no literature existed pertaining to the effective management of jet lag and travel fatigue in athletes. Therefore, it is critical that future work investigates the validity and efficacy of sleep and circadian interventions when athlete recovery following travel is insufficient.

We acknowledge potential limitations when accounting for circadian resynchronisation to the new time zone. League-wide open-sourced schedules provide game locations but not travel information, due to issues of confidentiality. Therefore, insights are based off a “blanket-rule” that all teams travelled to their next game destination immediately following their current game.

In summary, the current research examined the association between directional jet lag and performance and game outcome in the NBA over 10 regular seasons. Eastward jet lag was associated with impaired shooting, fewer rebounds, worse points differential and, ultimately, worse winning percentages for home (but not away) teams. In contrast, westward jet lag was not associated with performance or game outcome for home or away teams. Taken together, these findings offer support for the asymmetrical jet lag hypothesis—sleep and circadian disruption associated with advancing phase following eastward travel may have adverse consequences on performance in the NBA, particularly when recovery time is limited. With marginal gains being at the forefront of every elite program, our findings highlight an area that could play a significant role in who ends up top of the standings. Sports organisations should consider alternative scheduling and appropriately timed interventions to maximise recovery and performance of their athletes.

## Data Availability

Publicly available datasets were analyzed in this study. This data can be found here: www.basketball-reference.com.

## Footnotes

1 We did not examine associations between directional jet lag and mechanisms of the Optimal Timing Hypothesis as this hypothesis was not supported by the primary analyses in Aim 1.

2 We analysed points differential here, as binary outcomes like win vs loss can lack the sensitivity to uncover smaller effects when teams vary in ability (e.g., a superior team may be disadvantaged by jet lag but still beat an inferior team).

3 Full model results for all analyses are available in the Supplementary Material (S2).

## References

[B1] Arnett M. G. (2002). Effects of Prolonged and Reduced Warm-Ups on Diurnal Variation in Body Temperature and Swim Performance. J. Strength Cond. Res. 16 (2), 256–261. 10.1519/1533-4287(2002)016<0256:eoparw>2.0.co;2 PubMed Abstract | 10.1519/1533-4287(2002)016<0256:eoparw>2.0.co;2 | Google Scholar 11991779

[B2] Aschoff J. Hoffmann K. Pohl H. Wever R. (1975). Re-entrainment of Circadian Rhythms after Phase-Shifts of the Zeitgeber. Chronobiologia 2 (1), 23–78. PubMed Abstract | Google Scholar 1192905

[B3] Asher G. Sassone-Corsi P. (2015). Time for Food: The Intimate Interplay between Nutrition, Metabolism, and the Circadian Clock. Cell. 161 (1), 84–92. 10.1016/j.cell.2015.03.015 PubMed Abstract | 10.1016/j.cell.2015.03.015 | Google Scholar 25815987

[B4] Atkinson G. Reilly T. (1996). Circadian Variation in Sports Performance. Sports Med. 21 (4), 292–312. 10.2165/00007256-199621040-00005 PubMed Abstract | 10.2165/00007256-199621040-00005 | Google Scholar 8726347

[B5] Barnes S. (2021). "The Best Recovery You Could Possibly Get": Sleep, Rest, and the National Basketball Association. Sociol. Sport J. 38 (1), 16–25. 10.1123/ssj.2019-0111 10.1123/ssj.2019-0111 | Google Scholar

[B6] Burgess H. J. Crowley S. J. Gazda C. J. Fogg L. F. Eastman C. I. (2003). Preflight Adjustment to Eastward Travel:3 Days of Advancing Sleep with and without Morning Bright Light. J. Biol. Rhythms 18 (4), 318–328. 10.1177/0748730403253585 PubMed Abstract | 10.1177/0748730403253585 | Google Scholar 12932084 PMC1262683

[B7] Burgess H. J. Revell V. L. Molina T. A. Eastman C. I. (2010). Human Phase Response Curves to Three Days of Daily Melatonin: 0.5 mgVersus3.0 Mg. J. Clin. Endocrinol. Metabolism 95 (7), 3325–3331. 10.1210/jc.2009-2590 PubMed Abstract | 10.1210/jc.2009-2590 | Google Scholar PMC2928909 20410229

[B8] Burke T. M. Markwald R. R. Chinoy E. D. Snider J. A. Bessman S. C. Jung C. M. (2013). Combination of Light and Melatonin Time Cues for Phase Advancing the Human Circadian Clock. Sleep 36 (11), 1617–1624. 10.5665/sleep.3110 PubMed Abstract | 10.5665/sleep.3110 | Google Scholar 24179293 PMC3792377

[B9] Chapman D. W. Bullock N. Ross A. Rosemond D. Martin D. T. (2012). Detrimental Effects of West to East Transmeridian Flight on Jump Performance. Eur. J. Appl. Physiol. 112 (5), 1663–1669. 10.1007/s00421-011-2134-6 PubMed Abstract | 10.1007/s00421-011-2134-6 | Google Scholar 21874551

[B10] Clements E. Fowler P. M. Duffield R. (2021). “The Influence of Travel in the Home Advantage Effect,” in Home Advantage in Sport (New York, NY: Routledge), 118–130. 10.4324/9781003081456-14 10.4324/9781003081456-14 | Google Scholar

[B11] Colquhoun W. P. (1972). Aspects of Human Efficiency: Diurnal Rhythm and Loss of Sleep. London: English Universities Press. Google Scholar

[B12] Czeisler C. A. Duffy J. F. Shanahan T. L. Brown E. N. Mitchell J. F. Rimmer D. W. (1999). Stability, Precision, and Near-24-Hour Period of the Human Circadian Pacemaker. Science 284 (5423), 2177–2181. 10.1126/science.284.5423.2177 PubMed Abstract | 10.1126/science.284.5423.2177 | Google Scholar 10381883

[B13] Drust B. Waterhouse J. Atkinson G. Edwards B. Reilly T. (2005). Circadian Rhythms in Sports Performance-An Update. Chronobiology Int. 22 (1), 21–44. 10.1081/CBI-200041039 PubMed Abstract | 10.1081/CBI-200041039 | Google Scholar 15865319

[B14] Eastman C. I. Burgess H. J. (2009). How to Travel the World without Jet Lag. Sleep. Med. Clin. 4 (2), 241–255. 10.1016/j.jsmc.2009.02.006 PubMed Abstract | 10.1016/j.jsmc.2009.02.006 | Google Scholar 20204161 PMC2829880

[B15] Facer-Childs E. Brandstaetter R. (2015). The Impact of Circadian Phenotype and Time since Awakening on Diurnal Performance in Athletes. Curr. Biol. 25 (4), 518–522. 10.1016/j.cub.2014.12.036 PubMed Abstract | 10.1016/j.cub.2014.12.036 | Google Scholar 25639241

[B16] Facer-Childs E. R. Boiling S. Balanos G. M. (2018). The Effects of Time of Day and Chronotype on Cognitive and Physical Performance in Healthy Volunteers. Sports Med. - Open 4 (1), 47–58. 10.1186/s40798-018-0162-z PubMed Abstract | 10.1186/s40798-018-0162-z | Google Scholar 30357501 PMC6200828

[B17] Fowler P. M. Knez W. Crowcroft S. Mendham A. E. Miller J. Sargent C. (2017). Greater Effect of East versus West Travel on Jet Lag, Sleep, and Team Sport Performance. Med. Sci. Sports Exerc. 49 (12), 2548–2561. 10.1249/mss.0000000000001374 PubMed Abstract | 10.1249/mss.0000000000001374 | Google Scholar 28719491

[B18] Fox J. L. Stanton R. Sargent C. Wintour S.-A. Scanlan A. T. (2018). The Association between Training Load and Performance in Team Sports: A Systematic Review. Sports Med. 48 (12), 2743–2774. 10.1007/s40279-018-0982-5 PubMed Abstract | 10.1007/s40279-018-0982-5 | Google Scholar 30225537

[B19] Fullagar H. H. K. Duffield R. Skorski S. Coutts A. J. Julian R. Meyer T. (2015). Sleep and Recovery in Team Sport: Current Sleep-Related Issues Facing Professional Team-Sport Athletes. Int. J. Sports Physiology Perform. 10 (8), 950–957. 10.1123/ijspp.2014-0565 PubMed Abstract | 10.1123/ijspp.2014-0565 | Google Scholar 25756787

[B20] Haislop T. (2020). NBA Bubble, Explained: A Complete Guide to the Rules, Teams. schedule & more for Orlando games [Online]. Sporting News. Available: https://www.sportingnews.com/us/nba/news/nba-bubble-rules-teams-schedule-orlando/zhap66a9hcwq1khmcex3ggabo (Accessed February 3, 2022). Google Scholar

[B21] Harris A. R. Roebber P. J. (2019). NBA Team Home Advantage: Identifying Key Factors Using an Artificial Neural Network. PLOS ONE 14 (7), e0220630. 10.1371/journal.pone.0220630 PubMed Abstract | 10.1371/journal.pone.0220630 | Google Scholar 31365592 PMC6668839

[B22] Hoffman D. T. Dwyer D. B. Bowe S. J. Clifton P. Gastin P. B. (2020). Is Injury Associated with Team Performance in Elite Australian Football? 20 Years of Player Injury and Team Performance Data that Include Measures of Individual Player Value. Br. J. Sports Med. 54 (8), 475–479. 10.1136/bjsports-2018-100029 PubMed Abstract | 10.1136/bjsports-2018-100029 | Google Scholar 31242988

[B23] Janse van Rensburg D. C. Jansen van Rensburg A. Fowler P. Fullagar H. Stevens D. Halson S. (2020). How to Manage Travel Fatigue and Jet Lag in Athletes? A Systematic Review of Interventions. Br. J. Sports Med. 54 (16), 960–968. 10.1136/bjsports-2019-101635 PubMed Abstract | 10.1136/bjsports-2019-101635 | Google Scholar 32303523

[B24] Janse van Rensburg D. C. Jansen van Rensburg A. Fowler P. M. Bender A. M. Stevens D. Sullivan K. O. (2021). Managing Travel Fatigue and Jet Lag in Athletes: A Review and Consensus Statement. Sports Med. 51 (10), 2029–2050. 10.1007/s40279-021-01502-0 PubMed Abstract | 10.1007/s40279-021-01502-0 | Google Scholar 34263388 PMC8279034

[B25] Jones J. J. Kirschen G. W. Kancharla S. Hale L. (2019). Association between Late-Night Tweeting and Next-Day Game Performance Among Professional Basketball Players. Sleep. Health 5 (1), 68–71. 10.1016/j.sleh.2018.09.005 PubMed Abstract | 10.1016/j.sleh.2018.09.005 | Google Scholar 30670169

[B26] Leatherwood W. E. Dragoo J. L. (2013). Effect of Airline Travel on Performance: a Review of the Literature. Br. J. Sports Med. 47 (9), 561–567. 10.1136/bjsports-2012-091449 PubMed Abstract | 10.1136/bjsports-2012-091449 | Google Scholar 23143931

[B27] Leota J. Hoffman D. Mascaro L. Czeisler M. É. Nash K. Drummond S. P. A. (2021). Home Is where the Hustle Is: The Influence of Crowds on Effort and Home Advantage in the National Basketball Association. SSRN J. 10.2139/ssrn.3898283 10.2139/ssrn.3898283 | Google Scholar 36512468

[B28] Lewis P. Korf H. W. Kuffer L. Groß J. V. Erren T. C. (2018). Exercise Time Cues (Zeitgebers) for Human Circadian Systems Can Foster Health and Improve Performance: a Systematic Review. BMJ Open Sport Exerc Med. 4 (1), e000443. 10.1136/bmjsem-2018-000443 PubMed Abstract | 10.1136/bmjsem-2018-000443 | Google Scholar PMC6330200 30687511

[B29] Loat C. E. R. Rhodes E. C. (1989). Jet-Lag and Human Performance. Sports Med. 8 (4), 226–238. 10.2165/00007256-198908040-00003 PubMed Abstract | 10.2165/00007256-198908040-00003 | Google Scholar 2692117

[B30] Maheswaran R. Chang Y. H. Henehan A. Danesis S. (2012).Deconstructing the Rebound with Optical Tracking Data. Proceedings of the MIT Sloan Sports Analytics Conference. MIT, 1–7. Google Scholar

[B31] Manfredini R. Manfredini F. Fersini C. Conconi F. (1998). Circadian Rhythms, Athletic Performance, and Jet Lag. Br. J. Sports Med. 32 (2), 101–106. 10.1136/bjsm.32.2.101 PubMed Abstract | 10.1136/bjsm.32.2.101 | Google Scholar 9631214 PMC1756080

[B32] McHill A. W. Chinoy E. D. (2020). Utilizing the National Basketball Association's COVID-19 Restart "bubble" to Uncover the Impact of Travel and Circadian Disruption on Athletic Performance. Sci. Rep. 10 (1), 21827. 10.1038/s41598-020-78901-2 PubMed Abstract | 10.1038/s41598-020-78901-2 | Google Scholar 33311539 PMC7732833

[B33] Nutting A. W. Price J. (2017). Time Zones, Game Start Times, and Team Performance. J. Sports Econ. 18 (5), 471–478. 10.1177/1527002515588136 10.1177/1527002515588136 | Google Scholar

[B34] Recht L. D. Lew R. A. Schwartz W. J. (1995). Baseball Teams Beaten by Jet Lag. Nature 377 (6550), 583. 10.1038/377583a0 PubMed Abstract | 10.1038/377583a0 | Google Scholar 7566168

[B35] Reilly T. Atkinson G. Waterhouse J. (2000). in Exercise and Sport Science. Editors Garrett W.E.J. Kirkendall D.T. (Philadelphia: Lippincott Williams & Wilkins), 351–372.Chronobiology and Physical Performance Google Scholar

[B36] Reilly T. Waterhouse J. (2009). Sports Performance: Is There Evidence that the Body Clock Plays a Role? Eur. J. Appl. Physiol. 106 (3), 321–332. 10.1007/s00421-009-1066-x PubMed Abstract | 10.1007/s00421-009-1066-x | Google Scholar 19418063

[B37] Ribeiro H. V. Mukherjee S. Zeng X. H. T. (2016). The Advantage of Playing Home in NBA: Microscopic, Team-specific and Evolving Features. PLOS ONE 11 (3), e0152440. 10.1371/journal.pone.0152440 PubMed Abstract | 10.1371/journal.pone.0152440 | Google Scholar 27015636 PMC4807825

[B38] Roach G. D. Sargent C. (2019). Interventions to Minimize Jet Lag after Westward and Eastward Flight. Front. Physiol. 10 (927), 1–11. 10.3389/fphys.2019.00927 PubMed Abstract | 10.3389/fphys.2019.00927 | Google Scholar 31417411 PMC6684967

[B39] Roenneberg T. Kantermann T. Juda M. Vetter C. Allebrandt K. V. (2013). “Light and the Human Circadian Clock,” in Circadian Clocks. Editors Kramer A. Merrow M. (Berlin, Heidelberg: Springer Berlin Heidelberg), 311–331. 10.1007/978-3-642-25950-0_13 10.1007/978-3-642-25950-0_13 | Google Scholar 23604485

[B40] Roy J. Forest G. (2018). Greater Circadian Disadvantage during Evening Games for the National Basketball Association (NBA), National Hockey League (NHL) and National Football League (NFL) Teams Travelling Westward. J. Sleep. Res. 27 (1), 86–89. 10.1111/jsr.12565 PubMed Abstract | 10.1111/jsr.12565 | Google Scholar 28568314

[B41] Schmidt C. Collette F. Cajochen C. Peigneux P. (2007). A Time to Think: Circadian Rhythms in Human Cognition. Cogn. Neuropsychol. 24 (7), 755–789. 10.1080/02643290701754158 PubMed Abstract | 10.1080/02643290701754158 | Google Scholar 18066734

[B42] Seo D. Y. Lee S. Kim N. Ko K. S. Rhee B. D. Park B. J. (2013). Morning and Evening Exercise. Integr. Med. Res. 2 (4), 139–144. 10.1016/j.imr.2013.10.003 PubMed Abstract | 10.1016/j.imr.2013.10.003 | Google Scholar 28664065 PMC5481716

[B43] Simmons N. Mandal S. Paton B. Ahmed I. (2022). Are Circadian Rhythms a New Frontier in Athletic Performance? Curr. Sports Med. Rep. 21 (1), 5–7. 10.1249/jsr.0000000000000929 PubMed Abstract | 10.1249/jsr.0000000000000929 | Google Scholar 35018891

[B44] Singh M. Bird S. Charest J. Huyghe T. Calleja-Gonzalez J. (2021). Urgent Wake up Call for the National Basketball Association. J. Clin. Sleep Med. 17 (2), 243–248. 10.5664/jcsm.8938 PubMed Abstract | 10.5664/jcsm.8938 | Google Scholar 33112229 PMC7853218

[B45] Song A. Severini T. Allada R. (2017). How Jet Lag Impairs Major League Baseball Performance. Proc. Natl. Acad. Sci. U.S.A. 114 (6), 1407–1412. 10.1073/pnas.1608847114 PubMed Abstract | 10.1073/pnas.1608847114 | Google Scholar 28115724 PMC5307448

[B46] Sports Reference LLC (2017). Basketball-Reference.com - Basketball Statistics And History [Online]. Sports Reference LLC. Available: https://www.basketball-reference.com/ (Accessed February 3, 2022). Google Scholar

[B47] Stephan F. K. (2002). The “Other” Circadian System: Food as a Zeitgeber. J. Biol. Rhythms 17 (4), 284–292. 10.1177/074873040201700402 PubMed Abstract | 10.1177/074873040201700402 | Google Scholar 12164245

[B48] Takahashi T. Sasaki M. Itoh H. Sano H. Yamadera W. Ozone M. (1999). Re‐entrainment of Circadian Rhythm of Plasma Melatonin on an 8‐h Eastward Flight. Psychiatry Clin. Neurosci. 53 (2), 257–260. 10.1046/j.1440-1819.1999.00537.x PubMed Abstract | 10.1046/j.1440-1819.1999.00537.x | Google Scholar 10459704

[B49] Tucker R. Collins M. (2012). What Makes Champions? A Review of the Relative Contribution of Genes and Training to Sporting Success. Br. J. Sports Med. 46 (8), 555–561. 10.1136/bjsports-2011-090548 PubMed Abstract | 10.1136/bjsports-2011-090548 | Google Scholar 22535537

[B50] Walsh N. P. Halson S. L. Sargent C. Roach G. D. Nédélec M. Gupta L. (2021). Sleep and the Athlete: Narrative Review and 2021 Expert Consensus Recommendations. Br. J. Sports Med. 55 (7), 356–368. 10.1136/bjsports-2020-102025 10.1136/bjsports-2020-102025 | Google Scholar 33144349

[B51] Waterhouse J. Edwards B. Nevill A. Carvalho S. Atkinson G. Buckley P. (2002). Identifying Some Determinants of “Jet Lag” and its Symptoms: a Study of Athletes and Other Travellers. Br. J. Sports Med. 36 (1), 54–60. 10.1136/bjsm.36.1.54 PubMed Abstract | 10.1136/bjsm.36.1.54 | Google Scholar 11867494 PMC1724441

[B52] Waterhouse J. Reilly T. Atkinson G. (1997). Jet-lag. Lancet 350 (9091), 1611–1616. 10.1016/S0140-6736(97)07569-7 PubMed Abstract | 10.1016/S0140-6736(97)07569-7 | Google Scholar 9393352

[B53] Wehrens S. M. T. Christou S. Isherwood C. Middleton B. Gibbs M. A. Archer S. N. (2017). Meal Timing Regulates the Human Circadian System. Curr. Biol. 27 (12), 1768–1775. 10.1016/j.cub.2017.04.059 PubMed Abstract | 10.1016/j.cub.2017.04.059 | Google Scholar 28578930 PMC5483233

[B54] White M. H. Sheldon K. M. (2014). The Contract Year Syndrome in the NBA and MLB: A Classic Undermining Pattern. Motiv. Emot. 38 (2), 196–205. 10.1007/s11031-013-9389-7 10.1007/s11031-013-9389-7 | Google Scholar

[B55] Winter W. C. Hammond W. R. Green N. H. Zhang Z. Bliwise D. L. (2009). Measuring Circadian Advantage in Major League Baseball: A 10-Year Retrospective Study. Int. J. Sports Physiology Perform. 4 (3), 394–401. 10.1123/ijspp.4.3.394 PubMed Abstract | 10.1123/ijspp.4.3.394 | Google Scholar 19953826

[B56] Worthen J. B. Wade C. E. (1999). Direction of Travel and Visiting Team Athletic Performance: Support for a Circadian Dysrhythmia Hypothesis. J. Sport Behav. 22 (2), 279–287. Google Scholar

[B57] Yamazaki S. Numano R. Abe M. Hida A. Takahashi R.-i. Ueda M. (2000). Resetting Central and Peripheral Circadian Oscillators in Transgenic Rats. Science 288 (5466), 682–685. 10.1126/science.288.5466.682 PubMed Abstract | 10.1126/science.288.5466.682 | Google Scholar 10784453

[B58] Youngstedt S. D. Elliott J. A. Kripke D. F. (2019). Human Circadian Phase-Response Curves for Exercise. J. Physiol. 597 (8), 2253–2268. 10.1113/JP276943 PubMed Abstract | 10.1113/JP276943 | Google Scholar 30784068 PMC6462487

[B59] Zacharko M. Konefał M. Radzimiński Ł. Chmura P. Błażejczyk K. Chmura J. (2020). Direction of Travel of Time Zones Crossed and Results Achieved by Soccer Players. The Road from the 2018 FIFA World Cup to UEFA EURO 2020. Res. Sports Med. 30, 145–155. 10.1080/15438627.2020.1853545 PubMed Abstract | 10.1080/15438627.2020.1853545 | Google Scholar 33251863

