# Supplementary material for: Eastward Jet Lag is Associated with Impaired Performance and Game Outcome in the National Basketball Association
Source: Front Physiol. 2022 Jun 16;13:892681. doi: 10.3389/fphys.2022.892681 (PMC9245584; doi:10.3389/fphys.2022.892681)
Supplement: Supplementary file 1 [file DataSheet1.pdf]

Supplementary Material - *Eastward Jet Lag is Associated with Impaired Performance and Game Outcome in the National Basketball Association*

S1. Randomly selected example of one month of an NBA team's game schedule and assigned jet lag.

GAME SCHEDULE FOR THE LOS ANGELES CLIPPERS IN MARCH 2017.						
**SUN**	**MON**	**TUE**	**WED**	**THU**	**FRI**	**SAT**
			1 HOU<br>PT NONE	2	3 MIL<br>CT 1 EW	4 CHI<br>CT NONE
5	6 BOS<br>PT 1 WW	7	8 MIN<br>CT 1 EW	9 MEM<br>CT NONE	10	11 PHI<br>PT 1 WW
12	13 UTA<br>MT NONE	14	15 MIL<br>PT NONE	16 DEN<br>MT 1 EW	17	18 CLE<br>PT NONE
19	20 NYK<br>PT NONE	21 LAL<br>PT NONE	22	23 DAL<br>CT 1 EW	24	25 UTA<br>PT 1 WW
26 SAC<br>PT NONE	27	28	29 WAS<br>PT NONE	30 PHO<br>MT* NONE	31	

Note. Game schedule for the Los Angeles Clippers in March 2017. Calendar cells are divided into quadrants. Top left quadrant indicates the date in March 2017. Top right quadrant indicates the Los Angeles Clippers opponent. Bottom left quadrant indicates the time zone the game was played in (PT: Pacific; MT: Mountain; CT: Central; ET: Eastern). Bottom right quadrant indicates the jet lag value assigned to the Los Angeles Clippers for each game as a result of travel and rest days (WW: westward jet lag; EW: Eastward jet lag; none: no jet lag). Days of home games are shaded blue; days of away games are shaded red; days without games are unshaded.

*The state of Arizona does not observe daylight savings. Therefore, when the Los Angeles Clippers played the Phoenix Suns in Phoenix, Arizona on March 30, 2017, the time in Arizona was equal to the time in PT.

S2. Full model results for analyses reported in the manuscript.

Model	Fixed Effects	Estimate	Standard Error	df	T value	P value
Figure 2A	Intercept	0.57	0.02	38.78	24.31	< .001
	West. jet lag	0.009	0.02	11354.36	0.43	.67
	East. jet lag	-0.05	0.02	11444.23	-1.96	.05
Figure 2B	Intercept	2.53	0.69	39.49	3.65	< .001
	West. jet lag	0.41	0.58	11217.50	0.71	.48
	East. jet lag	-1.57	0.65	11440.97	-2.44	.01
Figure 3A	Intercept	2.52	0.70	39.47	3.62	< .001
	1 hr East.	-1.23	0.68	10822.89	-1.81	.07
	2 hr East.	-4.71	1.93	10805.60	-2.45	.01
Figure 3B	Intercept	2.48	0.70	53.66	3.52	.001
	No travel	-0.13	0.47	9872.12	-0.03	.98
	East. jet lag	-1.58	0.64	9854.18	-2.47	.01
Figure 4A	Intercept	1.06	0.36	40.42	2.96	.005
	West. jet lag	-0.07	0.38	11122.92	-0.188	.85
	East. jet lag	-1.46	0.43	11454.14	-3.38	< .001
Figure 5A	Intercept	0.01	0.004	54.23	2.64	.01
	West. jet lag	0.004	0.004	11405.68	0.946	.34
	East. jet lag	-0.01	0.004	1141.98	-2.848	.004

Note. Results of mixed linear models for home games including team, opponent, and game time as random effects. Fixed effects of specific models reported in the caption of the corresponding figure in the manuscript.
